# Raoultella Planticola: Bacteremia and Sepsis in a Patient with Cirrhosis

**DOI:** 10.7759/cureus.1508

**Published:** 2017-07-24

**Authors:** Michael R Povlow, Jaime Carrizosa, Adriana Jones

**Affiliations:** 1 Medical Student, University of Central Florida College of Medicine; 2 Infectious disease, University of Central Florida College of Medicine

**Keywords:** sepsis, bacteremia, raoultella planticola, spontaneous bacterial peritonitis, cirrhosis, alcohol

## Abstract

This case report describes a 66-year-old male who presented to the emergency department with sepsis and jaundice. He reported that he had been eating seafood at local restaurants for the past two weeks. The medications that he was taking at the time of admission included esomeprazole (proton pump inhibitor (PPI)), which may have contributed to his infection with Raoultella planticola. In addition, the patient had a prior medical history of alcoholic liver cirrhosis, which may have contributed to the bacteremia based on his immunocompromised status.

## Introduction

Raoultella planticola is a gram negative rod, previously classified in the Klebsiella family [1]. It has been reported as a cause of upper gastrointestinal (GI) infection, pancreatitis, cholangitis, bacteremia, and sepsis following the consumption of seafood [2-5]. The current literature discusses the risk factors for human infection with this bacteria, which include an immunocompromised state and liver cirrhosis. Heavy alcohol use may be another major risk factor, which could be important for determining at-risk populations. 

## Case presentation

A 66-year-old male presented to the emergency department with two days of fever, chills, generalized weakness, and anuria. He had a history of alcoholic cirrhosis, chronic kidney disease, and Barrett’s esophagus. The patient reported drinking alcohol every day. The patient's relevant medications included esomeprazole (40 mg) and furosemide (40 mg).

The vital signs on presentation were: temperature: 97.6 F; heart rate: 89; respiration: 18; and blood pressure: 60/27. His physical exam was significant for jaundice, right upper quadrant pain, and abdominal distension.

Pertinent lab results on admission included a white blood cell (WBC) count of 7.4x10^9^/L, a hemoglobin level of 6.9 g/dL, a platelet count of 70x10^9^/L, an international normalized ratio (INR) of 6.54, and a partial thromboplastin time of 54.7 seconds. His serum lactic acid was 7.55, procalcitonin was 8.9, and bilirubin was 13.2 total. Aspartate aminotransferase/alanine aminotransferase was 203/70 with a blood urea nitrogen/creatinine of 55/4.7. Blood cultures were obtained.

A computed tomography (CT) scan of the abdomen showed a cirrhotic liver with extensive portosystemic varices and a large right pleural effusion. An ultrasound of the abdomen showed findings consistent with liver cirrhosis, a large pleural fluid collection, and a thickening of the gallbladder wall.

Initial treatment was started with piperacillin/tazobactam for a broad spectrum coverage of infection. Blood cultures reported the growth of a gram negative rod identified as Raoultella planticola. The patient was switched to ceftriaxone based on sensitivities and prior literature on the treatment of this infection [4]. A thoracentesis was performed due to hydrothorax from the ascites, and 1800 milliliters of serosanguinous fluid was removed and analyzed. The fluid tested was consistent with an effusion secondary to spontaneous bacterial peritonitis and was the suspected cause of sepsis. Blood cultures taken two days after the initial set showed no bacterial growth and the patient had begun recovering from sepsis. Before discharge, the patient was counseled on his alcohol intake and seafood intake, both risk factors for infection by Raoultella planticola.

## Discussion

In this report, the patient stated that he had frequently consumed seafood in the weeks prior to the development of illness. His presentation suggested a picture of spontaneous bacterial peritonitis (SBP) with bacteremia in the presence of decompensated liver disease, cirrhosis, and massive ascites with the development of a large pleural effusion. The analysis of the pleural fluid did not show evidence of the most likely bacteria because of the early administration of appropriate antibiotics; however, the blood cultures taken two days earlier did show evidence of Raoultella planticola. There was no evidence of direct pulmonary infection. Unfortunately, the ascitic fluid was not examined.

There is a possible association of Klebsiella infection and Raoultella with alcohol abuse because of the colonization of the GI tract and the use of proton pump inhibitors (PPIs) in this setting [2-3,6]. Studies have shown that alcohol inhibits cytokines, which leads to a decrease in the host's defenses against bacteria, including Klebsiella [7]. Klebsiella and Raoultella are closely related bacteria [4] and, as a result, it is plausible that they would share some of these cytokine-releasing features, making alcoholics more prone to infection.

Ceftriaxone has shown excellent activity against Raoultella. In this case, the patient recovered well from this episode of sepsis. Some caution has to be taken because of recent reports of carbapenem-resistant strains [8].

## Conclusions

Raoultella planticola is an uncommon bacterium that is infectious. It is easy to be misidentified as the Klebsiella species during clinical laboratory testing. This is probably due to its prior history of being associated with Klebsiella and its more recent branching off as its own species. Now that we understand that this may be a potential cause of infection, especially in high-risk patients with many comorbidities, such as alcoholism, liver cirrhosis, PPI use, and immunocompromised state, it needs to be considered as a possible cause of the infection. This is especially true in patients with a recent history of seafood ingestion, as this has been identified as a possible source. Being able to identify the organism properly will help with more targeted treatments. It is also important to counsel patients on diet and alcohol intake to prevent future infections. In other reports, and in this case, ceftriaxone has been an effective choice for treatment, although individual sensitivities should be performed on a case-by-case basis to achieve the best results and decrease resistance.
